# Media-Based Post-Event Impact Analysis of the 2021 Heat Dome in Canada

**DOI:** 10.1177/11786302241276669

**Published:** 2024-09-05

**Authors:** Emily J Tetzlaff, Nicholas Goulet, Melissa Gorman, Gregory RA Richardson, Paddy M Enright, Sarah B Henderson, Glen P Kenny

**Affiliations:** 1 Human and Environmental Physiology Research Unit, School of Human Kinetics, Faculty of Health Sciences, University of Ottawa, Ottawa, ON, Canada; 2 Heat Division, Climate Change and Health Office, Health Canada, Ottawa, ON, Canada; 3 Behavioural and Metabolic Research Unit, School of Human Kinetics, Faculty of Health Sciences, University of Ottawa, Ottawa, ON, Canada; 4 Environmental Health Services, British Columbia Centre for Disease Control, Vancouver, BC, Canada; 5 Clinical Epidemiology Program, Ottawa Hospital Research Institute, Ottawa, ON, Canada

**Keywords:** Extreme heat event, heat wave, climate change, impact analysis, risk communication

## Abstract

The unprecedented 2021 Heat Dome caused wide-ranging and long-lasting impacts in western Canada, including 619 confirmed heat-related deaths in British Columbia, a doubling of emergency medical calls, increased hospitalisations, infrastructure failures and stress on plants and animals. However, such varied socio-economic consequences of extreme heat can be challenging to capture using a single post-event analysis method. Therefore, there is a need to explore alternative approaches and data sources. Using the 2021 Heat Dome as a case study, a post-event analysis using online news media articles (n = 2909) from 5 subscription news databases and a grey literature search was conducted to identify the socio-economic impacts of the extreme heat event in Canada. The articles reported a wide range of effects to the natural environment (n = 1366), social infrastructure and services (n = 1121), human health (n = 1074), critical infrastructure (n = 988) and the private sector (n = 165). The media-based post-event analysis captured various impacts, some of which have not been identified through other data sources and approaches. Overall, we show that media analysis can complement traditional post-event analysis methods and provide additional perspectives to governments and public health and safety officials.

## Introduction

Extreme heat events (EHEs) have substantially impacted public health.^1-3^ For example, thousands of excess deaths have been reported during EHEs around the world,^4^ including the 2003 European EHE,^5,6^ the 2013 EHE in central and eastern China, Japan and Korea,^7^ the 2017 EHE that affected countries across Europe,^8^ the unprecedented EHEs over northeast Asia in 2018^9,10^ and western North America in 2021 (termed ‘2021 Heat Dome’).^11^ EHEs also affect morbidity, with evidence of higher emergency department admissions,^12^ as well as increased ambulance calls and hospital admissions due to mental and behavioural disorders.^13,14^ Accompanying the serious consequences of heat on human health are other socio-economic impacts across multiple areas,^15,16^ including infrastructure failures (e.g., transportation, electricity transmission), exacerbated drought conditions and increased stress on plants^17-21^ and animals.^22-24^ EHEs may also co-occur with other environmental disruptions such as wildfires, high levels of ground-level ozone and droughts.^25-29^ These environmental disruptions can subject individuals and communities to profound socio-economic ramifications for prolonged periods as they evolve.^30-32^

To identify the diverse socio-economic impacts of EHEs, different organisations – including coroners and medical examiners, healthcare providers, governments, public health authorities, academics, non-governmental organisations and groups within the private sector – have conducted post-event analyses, often known as after-action reviews. The purpose of such investigations is to better understand the scope of impacts, identify lessons learned,^33^ and inform future efforts to communicate risks and develop interventions.^3,34,35^ However, these investigations are typically directed by the priority of the agency conducting the analysis and thus consider only the most relevant qualitative and quantitative data sources. For example, a coroner’s service would look at human mortality data, but is unlikely to explore impacts on wildlife. As a result, post-event analyses frequently fail to document the interconnectedness of outcomes across multiple impact areas and sectors.^36^ Therefore, there is a need to consider alternative post-event analysis approaches that can capture these complexities more comprehensively.

Recent studies have begun using alternative data sources for analysing the implications of EHEs.^37,38^ For example, Pamukcu et al^39^ and Wu^40^ analysed non-emergency service call data, and Spruce^41^ investigated social media posts for EHE-related impacts. Furthermore, Painter et al^42^ assessed a collection of traditional news media to understand the 2019 European EHE in France, Germany, the Netherlands and the United Kingdom. This latter approach may be beneficial for capturing effects across multiple sectors, because content published by the media draws on varied sources, captures consequences across different impact areas, and becomes available quickly compared with government inquiries and formal reviews.^43^ Further, the media can provide ongoing information after the EHE ends, which may help to identify both short- and longer-term lagging effects across sectors.^3^ Currently, only a few analyses have evaluated media articles published about EHEs, including a study looking at the role of the media in increasing the visibility of climate change in reporting on the 2021 Heat Dome,^44^ as well as investigations into the intersection of the 2021 Heat Dome with the COVID-19 pandemic^45^ and workplace health and safety implications.^46^ However, to our knowledge, no studies have systematically used media articles as the data source for conducting a post-event analysis of heat-related effects across multiple impact areas and sectors.

To help fill this gap, we conducted a post-event analysis of the 2021 Heat Dome using online news media as the data source. Analysing the several thousand news media articles published during and after the 2021 Heat Dome in Canada allowed us to identify the wide-ranging effects of the EHE across multiple impact areas, including the natural environment, social infrastructure and services, human health, critical infrastructure and the private sector. Given the extreme nature of the event and known consequences documented in other analyses e.g.,^47-49^ the 2021 Heat Dome provided an ideal case study to explore the evolving, wide-ranging and long-lasting impacts of EHEs. Thus, our study offers a novel, complementary approach to traditional post-event analysis and helps to identify emergent themes that may improve future interventions, such as risk communication. This may help to build community resilience and support societies in responding to EHEs in Canada and globally.

## Materials and Methods

### Case context: The 2021 Heat Dome

Between June 24th and July 4th, 2021, the unprecedented 2021 Heat Dome affected millions of people in western Canada and the United States. The 2021 Heat Dome impacted a vast area, including northern California, Idaho, western Nevada, Oregon and Washington in the United States and British Columbia (B.C.), Alberta, Saskatchewan, Northwest Territories and the Yukon in Canada.^48-50^ Over the course of the nearly 2-week period, the 2021 Heat Dome broke more than 103 all-time heat records, including Canada’s highest ever measured temperature (49.6°C Lytton, B.C. on June 29th, 2021), and the highest temperature ever recorded north of latitude 60° (39.9°C Fort Smith, Northwest Territories on June 30th, 2021).^48-50^ The 2021 Heat Dome led to a series of impacts locally, regionally and nationally, including at least 619 heat-related deaths in B.C., as well as a significant increase in hospitalisations and emergency calls.^51^ This event affected critical community infrastructure, services, businesses and the environment, including the mass die-off of marine life, devastated crop and livestock yields, increased wildfires throughout the province, and flooding from rapid snow and glacier melt.^49,52^ Further, due to the coinciding COVID-19 pandemic^2,45^ and ongoing drug overdose emergency in Canada,^53^ the resilience of the affected communities was impacted by the high demands already placed on health and social service systems that otherwise might have been mobilised to reduce the health burden of the 2021 Heat Dome.^11^

### Search strategy

We systematically reviewed digitised media content about the 2021 Heat Dome in Canada, including newspaper articles and text transcripts of radio and television broadcasts. To minimise reliance on prestige press (e.g., The Globe and Mail, Toronto Star, The Province) and limit outlet bias,^54^ we used 5 subscription news databases (ProQuest Canadian Major Dailies, Business Source Elite, NewsDesk, Factiva and Eureka). These databases provide access to current content and significant backfiles for Canada’s highest circulating national and regional newspapers, as well as smaller outlets, in full-text format. The search strategy was developed in consultation with a Research Librarian, and the final search underwent PRESS review before database translation (see Supplemental File in Ref.^55^ for more details). For all searches, the content was limited to English and French articles published within Canada between June 1st, 2021 and February 26th, 2022. This study excluded all social media posts and content without verbatim text transcriptions (e.g. audio- and video-only content).

**Figure 1.**
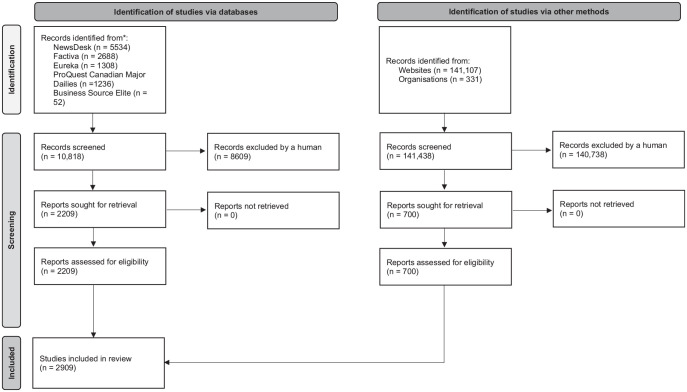
PRISMA-ScR flow diagram of search and study selection process. Abbreviation: PRISMA-ScR, Preferred Reporting Items for Systematic Reviews and Meta-analyses for Scoping Reviews.

In addition to the database searches, a list of targeted public and non-profit organisation websites was created for each province and territory in Canada, along with pan-Canadian sites, to identify relevant news/press release statements from the organisations. Based on the geographic impact of the 2021 Heat Dome, the priority focus for detailed searching was on the western provinces (B.C., Alberta, Saskatchewan and Manitoba). Sectors included health, environment, agriculture, infrastructure, housing, labour, safety, energy, school boards, municipalities and Indigenous communities. For the remaining provinces and territories (n = 9), a simplified search was completed for health, agriculture, housing and labour. For each targeted website (n = 997), the terms ‘heat’ and ‘2021’ were entered into the search function. When no search function was available, the researchers (E.J.T. and N.G.) performed a general site search targeting the homepage, news, newsletters and publication/resource tabs. Lastly, a Google search was performed for each province and territory to ensure coverage of publicly accessible online news sources. The Google searches were completed until the display notice: ‘*In order to show you the most relevant results, we have omitted some entries very similar to the X already displayed*’ was reached (see Supplemental File in Ref.^55^ for more details; Figure 1).

### Article selection process

The articles were uploaded to Zotero (Release 6.0, Corporation of Digital Scholarship), a digital citation tool. Similar versions of articles released to multiple news sources were identified, and duplicates were removed. Articles written in French were identified and sent for professional translation to English. The translated documents were then checked for accuracy by a bilingual research team member (N.G.). Due to the large dataset size, full-text review co-occurred with content coding to ensure that the data included only Canadian-produced news articles and that the text primarily focused on the Canadian context.

### Building and refining the coding frame

Based on a preliminary review of the articles collected, 5 impact areas were pre-identified with 20 related themes: (1) Natural Environment, including (a) natural systems, (b) natural resources, and (c) animals; (2) Social Infrastructure and Services, including (a) healthcare, (b) transportation services, (c) education, (d) community and social supports, (e) sports and recreation, (f) arts, culture and tourism, (g) crime, corrections and justice, and (h) waste management; (3) Human Health, including (a) mortality and (b) physical, and (c) mental health consequences; (4) Critical Infrastructure, including (a) food systems (agriculture and associated supply chain), (b) community water supply, (c) energy, (d) transportation infrastructure, (e) communication, and (f) commercial buildings; and (5) Private Sector, meaning businesses. In addition to the pre-identified impact areas (n = 5) and themes (n = 20), we coded inductively (data-driven) for additional concepts to assist in identifying more specific or granular topics of interest. Similar pattern identification was completed for all impact areas and themes through iterative coding. For our analysis, coded data extracts (e.g., words, sentences, paragraphs) were assigned to as many different impact areas and concepts as necessary to describe them. As such, the count values presented in the narrative results and tabular reporting do not equal, and can sometimes exceed, the summative total in which it is nested.

Lastly, any content in the media articles related to direct, compounding, or cascading effects was captured for each impact area and theme. This included direct outcomes associated with the 2021 Heat Dome, such as people experiencing heat stroke. It also included impacts caused by cascading events entirely or partially attributed to the EHE, such as wildfires that caused infrastructure or environmental damage, and indirectly related outcomes, such as high ground-level ozone concentrations leading to respiratory health effects. Further, we also captured consequences related to compounding events that intersected with the EHE, or were amplified by the heat, such as existing drought made worse by the EHE and its effects on crops.^27,31,56^

### Piloting and primary coding

Once the structure of the coding frame was developed, the whole research team reviewed the codes to determine if any residual categories should be identified and amended. The team then sought agreement on definitions, positive indicators, decision rules and textual examples. All files and the codebook were then uploaded to NVivo (Release 1.6.2, QSR International), and a trial coding exercise was completed by 2 independent coders (E.J.T. and N.G.) on a sample of articles (n = 500). A coding comparison query conducted in NVivo^57^ was also used to ensure the consistency (reliability) and validity of category definitions.^58^ The coders achieved a kappa coefficient of 0.84 for this analysis, indicating ‘very good/excellent’ agreement.^57,59^ All articles were then fully coded.

### Data analysis

After coding was complete, the analysis was conducted in 3 steps. First, the characteristics of the included documents and extracted data were analysed using a series of NVivo query functions. Next, a qualitative content analysis method for systematically describing the meaning of the data was used.^61^ The authors then met to discuss the data and agree on the broader themes and concepts.

## Results

**Figure 2.**
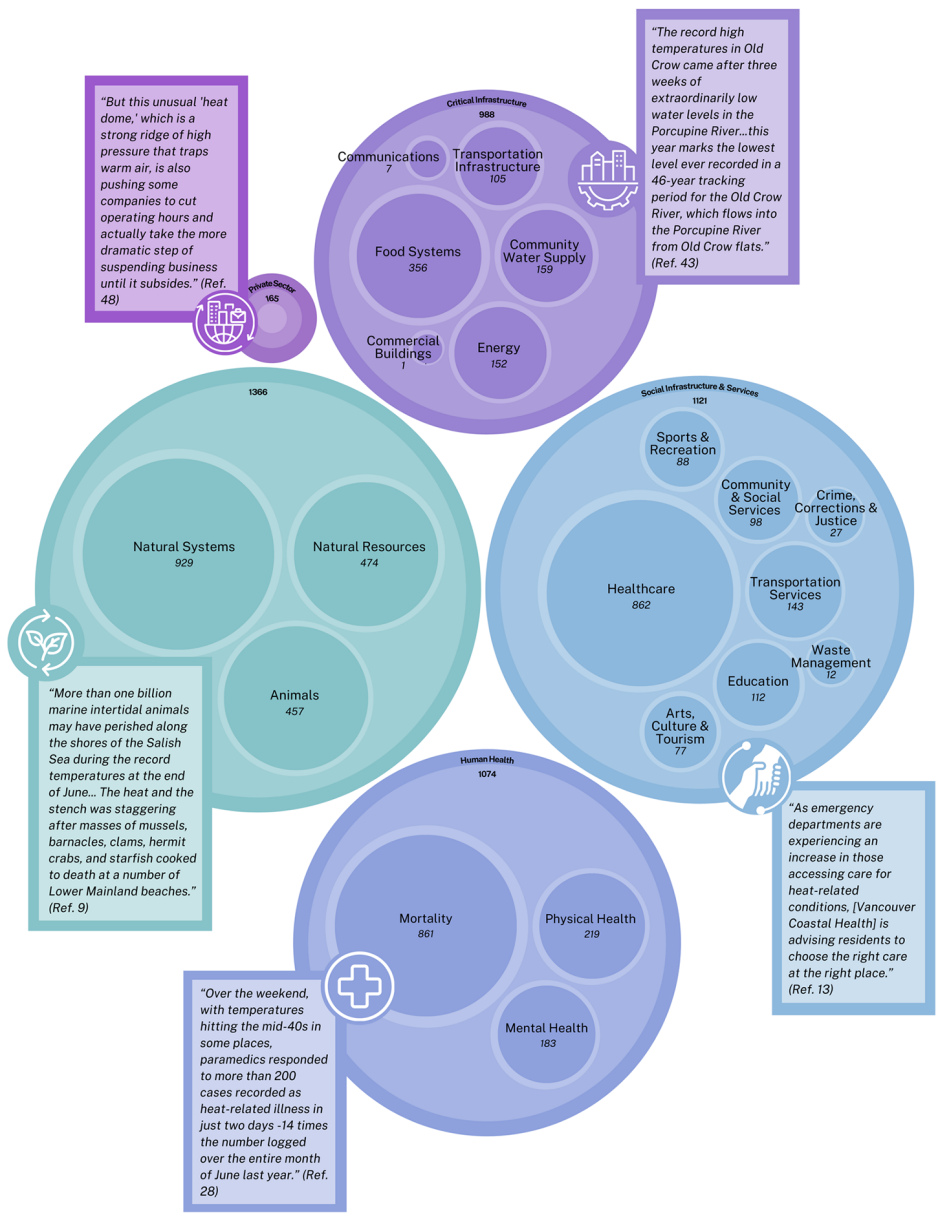
Visual depiction of the themes and concepts identified in the media post-event impact analysis of the 2021 Heat Dome. Each outer circle represents a theme identified in the media articles, with the nested circles representing the concepts identified. The size of the circle represents the dominance of the theme or concept.

We included 2909 articles on the 2021 Heat Dome from Canadian news outlets. These articles included content related to the 5 impact areas: (1) Natural Environment (n = 1366); (2) Social Infrastructure and Services (n = 1121); (3) Human Health (n = 1074); (4) Critical Infrastructure (n = 988); and the (5) Private Sector (n = 165). Themes and concepts are provided descriptively below and visually in Figure 2 within each impact area based on evidence found within the news coverage analysed and are oriented from most to least dominant based on frequency. Thus, we are presenting the findings as reported and, therefore, are not commenting on the accuracy or validity of the media reports.

### Impacts on the natural environment

Among the media articles, 1366 reported on the natural environment and the 2021 Heat Dome, including its effects on natural systems, natural resources and animals (Supplemental Table S1). The most frequently reported impact related to the natural environment was the connection between extreme heat and natural systems (n = 929) such as wildfires, firestorms (occurs when heat from a wildfire creates its own wind system), lightning strikes, thunderstorms, hailstorms, tornados and downbursts. The reported impacts on the natural environment also extended to vegetation and plants, including seaweed, kelp, plankton, soil, deer browse, fungi and ocean spray. As a result of the impacts on the natural environment, various natural hazards and environmental health risks were referenced (n = 474), such as flooding, melting of ice caps, glaciers and snow, mudslides and landslides, atmospheric rivers, heavy rainfall, cyclones and avalanches. Air quality concerns were reported due to the elevated ground-level ozone from the EHE, smoke from the wildfires sparked during the 2021 Heat Dome, and the movement/stagnancy of the air. Articles that reported air pollution impacts frequently also mentioned the accompanying health implications because the harmful, constantly changing mixture of particles and gasses released in wildfire smoke can irritate the airway and can cause severe problems for people with asthma, chronic obstructive pulmonary disorder, lung cancer or heart problems. During and after the 2021 Heat Dome, polluted air was reported to have led to a spike in patients suffering respiratory issues. The poor air quality also impacted social infrastructure and services, with officials closing some public facilities and cancelling certain events to reduce population exposure. Similarly, some workplaces and occupations were affected by the effects on the environment, including postal workers and people working on field-crop and livestock farms. Likewise, some field-crop farmers reported that wildfire smoke blocked the UV light needed for plant photosynthesis, exacerbating crop damage.

The media reported that the 2021 Heat Dome impacted many animals (n = 457). The articles portrayed stories of pets being exposed to extreme temperatures indoors (in homes and cars) and outdoors, with numerous cases reporting pets succumbing to heat stroke or other co-morbidities exacerbated by the heat. As well, the heat affected farm animals by causing feed shortages and dehydration. As a result, reports suggest farmers experienced production impacts because farm animals died from the heat or were sent to slaughter. For example, chickens and other domesticated fowl housed in barn facilities were exposed to very high indoor temperatures, placing birds under stress and causing them to produce smaller eggs or stop laying eggs at all. The media also described the additional challenges faced by farm animals due to the inhalation of smoke caused by the subsequent wildfires, which caused lower birthrates and reduced milk production for cows. Similarly, the extreme temperatures led to a mass die-off of wild aquatic animals, including fish, molluscs, crustaceans, sea anemones and sea mammals. Most of these consequences were attributed to the extreme heat, high tide and the summer solstice. Articles discussed the implications of this mass wild aquatic animal die-off and how these creatures play critical roles in the ecosystem.

There were reports of heat impacts on insects and arthropods (n = 59), with many articles noting that insect populations were found to thrive in dry conditions. Lifecycles were accelerated, allowing for rapid development and maturation earlier in the season for insects such as ants, flies and yellowjacket wasps, with some species doubling their colony sizes. In addition, the persistently high temperatures created the perfect conditions to support larger populations of many insects, such as aphids, further increasing the buildup and ability to produce multiple generations within the year. As a result of the increase in insect populations, numerous secondary repercussions were also cited, such as the consequences to farmers’ livelihoods in the prairies as grasshoppers damaged crops. Another related concern was the impact of insects on human health and well-being. For example, the media articles referenced public health concerns, describing how the extreme heat would complicate efforts to address the West Nile virus and Lyme disease because of hot, dry weather favouring mosquitoes and tick vectors. The media articles also reported cases of wild land animals experiencing heat stress, such as deer spotted with open mouths and panting in the heat. Other articles reported the lack of water for wild animals and the compounding risks this posed for the animals and their interactions with humans. For example, mountain goats were seen climbing down to busy highways searching for water on scorching asphalt roads, and black bears were forced closer to communities along Canada’s West Coast due to the devastated berry crops, leading to reports of increased human-bear interactions. Media reports related to captive wildlife – such as animals kept at research laboratories, zoos and reptile sanctuaries – suggest they were also affected by the 2021 Heat Dome. For example, reptile centres reported deaths of rodents, snakes and geckos due to the extreme heat. Lastly, articles discussed how the extreme heat impacted wild birds, with reports of many suffering from heat exhaustion and succumbing to the heat, especially nestlings, baby birds and juvenile birds.

### Impacts on social infrastructure and services

Among the media articles, 1121 reported the impact of the 2021 Heat Dome on social infrastructure and services in Canada (Supplemental Table S2). Many of these articles reported implications to the healthcare system (n = 862), including disruptions and overburden on pre-hospital and hospital services, coroner’s services, long-term care facilities and pharmacies. Public health services were also impacted, such as the cancellation or rescheduling of vaccination clinics for COVID-19. Public transportation services were also affected (n = 143), including disruption of services from infrastructure failures, reduction to services to protect equipment from extreme heat and prioritise passenger and driver safety, and shifts in service purpose to provide access to cooling centres rather than business as usual. As the start of the 2021 Heat Dome overlapped with the final days of the academic year for most school boards, repercussions were reported to the education system (n = 112), including elementary, secondary and post-secondary school/campus closures or modifications and the cancellation of school bus services. Likewise, the EHE affected aspects of sports and recreation in affected communities (n = 88), including the cancellation or closure of fitness and recreational activities/facilities, restrictions on park access hours and rescheduling of sports child/youth camps. Direct effects were also reported on park infrastructure, such as overheated playground equipment and melted park benches. In addition, the extreme heat impacted backcountry activities, including camping, fishing, hiking, climbing and bouldering; many jurisdictions banned campfires and fireworks.

During the 2021 Heat Dome, many impacts on tourism were reported (n = 77). For example, numerous community activities were postponed or cancelled, including Canada Day (national holiday) celebrations and activities at the Calgary Stampede (n = 98). Similarly, many museums and heritage sites were affected by the extreme heat and some locations reported repercussions on the number of site guests or longevity of park stays. In addition to the direct extreme heat effects, the subsequent wildfires that intersected with the 2021 Heat Dome further impacted museums and heritage sites. For example, two museums were burned due to the Lytton fire, one losing more than 1500 artifacts. There were also reports that crime, corrections and justice were affected (n = 27), such as the cancellation or postponement of assemblies, rallies, protests and eviction notices. There were also reports of a courthouse evacuation and closure as smoke poured from an overheated generator and the power went out. Lastly, it was reported that waste management was impacted (n = 12), including curbside collection cancellations and disruptions due to the extremely high temperatures and concerns for the health and safety of collection crews.

### Impacts on human health

The news coverage of the 2021 Heat Dome discussed impacts on human health (n = 1074; Supplemental Table S3). The effects ranged from the unprecedented death toll resulting from the heat (n = 861) to other physical health issues and chronic disease management. Media reports on the physical impacts of heat (n = 219) included content on heat-related illnesses, such as heat stroke and heat exhaustion, as well as increased hospitalisations due to dehydration and cardiac emergencies. Some articles discussed the effect of heat on the brain’s capacity to cope and adapt behaviourally, resulting in further adverse physical effects. The news coverage also often reported on how the heat triggered a wave of patients suffering worsening mental health conditions (n = 183), such as depression and anxiety, as well as the distress caused by seeing people die because of the EHE. This led to reports of symptoms of post-traumatic stress disorder, shame, guilt and grief because of peoples’ experiences and the uncertainty of surviving future EHEs. There was a particular emphasis on reporting the mental health impacts on agricultural and livestock producers and healthcare providers such as paramedics and coroners. There were reports of increased requests for occupational leave due to the stress and trauma from the job, as well as moral distress among healthcare workers due to the substantial morbidity and mortality experienced during the event. Beyond the immediacy of the event, the articles following the 2021 Heat Dome continued to discuss the mental health burden of changing climatic conditions, referring to eco- or climate-anxiety as an emergent mental health concern, as well as anxiety about electricity supply issues (power outages) during future EHEs and other extreme weather events.

### Impacts on critical infrastructure

The dry weather and record-setting heat also tested the capacity of critical utility infrastructure (n = 958; Supplemental Table S4). The articles emphasised that food producers, processors, distributors and retailers (n = 356), along with the communities they serve, were particularly impacted. Most articles reporting on this topic focussed on the considerable losses experienced by livestock producers and cattle ranchers, as well as the rising cost of feed prices due to the supply shortages experienced by grain and field-crop producers owing to the heat-induced lower harvest volumes in the Prairie provinces of Alberta, Saskatchewan and Manitoba. Processors and distributors, such as canning companies and bakeries, were also affected because crop losses led to low supply and the requirement to seek new suppliers. As a result of these impacts, the media reported on rises in grocery prices, projections of food shortages and concerns over food security across the country for more than 6 months post-event.

The heat also disrupted the natural water cycle (n = 159) and led to recorded summertime highs for water demand, creating substantial water management problems for residential, agricultural and industrial water users and significant ecosystem disruption. For residential users, the low water levels caused service interruptions, community-wide usage restrictions, closure or reductions of spray parks for cooling, water pressure issues due to pump system limitations, and, in some cases, complete shutdown of community water supplies. The exceptionally high residential water demand in many areas impacted water supplies for fire safety, with a few departments seeking augmented water supply from ponds and finding water levels below the intake. In addition, many articles illustrated how impacts to critical infrastructure like water access subsequently affected human health, the natural environment and social infrastructure. For example, in some communities, boil water advisories were issued due to low water levels in some reservoirs, which had consequences for the effectiveness of chlorine disinfection in removing or inactivating pathogens. The low water supply also affected leisure activities, including ending the recreational fishing season early and marinas refusing to fill water tanks for boaters. Adverse impacts on the ecosystem were observed, including negative outcomes for aquatic animals, including fish, due to very high-water temperatures combined with low flow conditions, dewatering of riffle habitats and disconnected side channels. It was also reported that the low- and high-water levels increased water temperatures, which accelerated permafrost melt in Canada’s North and destabilised land and water bodies, further impacting fish spawning, birds nesting and muskrat and caribou habitats. This had cascading implications on traditional harvesting and cultural practices, in turn affecting health, social well-being and Indigenous ways of life.

The 2021 Heat Dome increased energy demand (n = 152), which taxed power grids and resulted in system failures, outages and infrastructure damage, including melted power lines, transformer burnouts and submarine cables bulging and leaking. These damages caused individual users and larger industrial customers to reduce power use or conserve energy due to the EHE. Multiple impacts were reported in communities that experienced power outages, including closing designated cooling centres and extending hours longer to accommodate residents and farmers experiencing refrigeration system failures and spoilage.

As a result of the extreme heat and subsequent cascading weather events, transportation infrastructure in western Canada was impacted (n = 105). This included roadways and highways heaving and buckling, car windows and asphalt melting and drawbridges malfunctioning. Cascading natural hazards that happened later in the year, such as wildfires and flooding, further damaged highway infrastructure and train lines and also had widespread implications for the transportation networks. For example, milk shortages were experienced because the transportation routes remained blocked due to flooding and fires, with some farmers reporting pouring thousands of litres of milk down the drain because delivery trucks could not get through to the farms because of impassable roads. Heat and cascading event-related impacts on communication included extensive physical damage to communication towers (n = 7), which disrupted cellphone networks and made communication difficult in some areas. In addition to phone services, traditional mail services were also affected because postal outlets were physically damaged by the cascading events, and mail delivery was suspended due to the heat. Similarly, newspaper carriers in some areas were opting to wait until the evening to deliver the paper to avoid the heat, affecting the news coverage flow. Lastly, although only reported in 1 article, damage was experienced to a commercial building after tempered glass panels exploded in the heat (n = 1).

### Impacts on the private sector

Based on the reports circulated in the media, the 2021 Heat Dome impacted Canada’s private sector (n = 165; Supplemental Table S5). These diverse effects included work cancellations, stoppages, delays, or modifications to over 60 occupations and workplaces. These outcomes were also reported to have decreased work demand and resulted in a loss of income and caused concern for job security for some workers (e.g. agricultural pickers). Conversely, the 2021 Heat Dome was also reported to impact some workplaces positively, increasing work demand and economic gain. For example, electrical utility workers and air conditioner repair crews faced increased work due to the public need for mechanical cooling. There was also evidence that the implications to the private sector corresponded with those to human health, with some reports of heat-related illnesses and death identified among workers, as well as deterioration of worker mental health due to loss of income, job stability and security. The health consequences of heat strain were also reported to have diminished worker productivity, which subsequently impacted businesses.

## Discussion

We systematically reviewed and qualitatively analysed the content of thousands of news articles reporting on the 2021 Heat Dome to assess the novel application of a media-based post-event analysis method. This approach to studying the impacts discussed in the media demonstrates the far-reaching effects of the 2021 Heat Dome across multiple sectors, including the natural environment, social infrastructure and services, human health, critical infrastructure and the private sector. It also highlights outcomes of the EHE that may not typically be captured in post-event analyses or the peer-reviewed literature. Further, by using media as the data source for this post-event analysis we also gained insight into the selection of what content, or impacts are deemed newsworthy to share and therefore shaped what was presented to the public on this climate-driven extreme weather event. The following sections outline our key observations and share how a post-event media analysis can be used to complement traditional post-event analysis methods.

Post-event analyses of media articles provide a strategy for identifying multiple impact areas previously only uncovered by individual analysis of singular impact areas. For example, many of the outcomes identified in our media-based post-event analysis align with findings from previous studies of specific EHE outcomes, as well as recent publications on the 2021 Heat Dome.^61,62^ These include high human mortality rates, the risks of power outages in many areas, water shortages and transportation disruptions, as well as the implications on the ecosystem and industries. Within the media coverage, we also identified many references to the impact of the 2021 Heat Dome on the natural environment – including natural systems, natural resources and animals. These included short-term impacts such as animal mortality and plant die-off, as well as forecasted long-term effects such as loss of habitat. Both the short- and long-term impacts align with findings from recent studies on the 2021 Heat Dome’s effect on vegetation^61,63,64^ and related land and marine ecosystems.^49,65-67^ Similarly, the media prioritised human health content (n = 1074), aligning with findings in recent publications and investigations describing sizable heat-related morbidity and mortality outcomes.^1,11,68^ Further, mental health impacts were mentioned in many media articles (n = 183) using terms such as ‘eco-anxiety’ and ‘climate grief’ when communicating these concepts and integrating them with stories of lived experiences from those affected by the 2021 Heat Dome. This finding is corroborated by a study by Bratu et al,^47^ which identified a rise in mental health issues in B.C. following the 2021 Heat Dome. Therefore, our approach to post-event media analysis demonstrates its ability to complement traditional post-event analysis methods while offering the benefit of drawing on a data source – media articles – that reported impacts of the EHE in near-real time as events evolved.

In addition to previously reported findings, our study captured a range of effects related to the 2021 Heat Dome not previously or commonly seen in post-event analysis conducted using other data sources. For example, within the social infrastructure and services impact areas, we identified numerous reports about cancelled and disrupted services during the EHE, such as waste collection. This challenged the workforce due to rescheduling and staffing and created a public safety concern for regions where bears or other wildlife may have been attracted to accumulated waste. To our knowledge, such findings have not been identified in other EHE impact analyses. Another unique example is related to elevated water temperatures, leading to food poisoning or intestinal illness cases. For instance, it was reported that ‘*several people fell ill with food poisoning in B.C. after an unprecedented heat wave last month. Satellites recorded temperatures of 20°C a full 10 metre below the ocean’s surface – as a result, marine life died, and people got sick from either swimming recreationally or harvesting their shellfish during the heat wave*’.^48^ This quote among other examples, illustrates how the media reported on novel and cascading effects of the 2021 Heat Dome that crossed multiple sectors and systems and are less commonly seen in post-event reports and the peer-review literature. Therefore, media-based analysis can provide valuable insight into the critical outcomes of an EHE across multiple impact areas that may otherwise not be captured by conventional methods. Identifying multiple impact areas jointly supports a better understanding of the cascading and compounding impacts of EHEs, providing information that could underpin interventions across sectors.

We also identified specific subgroups that were disproportionally affected by the 2021 Heat Dome. For example, multiple community members with disabilities were interviewed by reporters to highlight the challenges they faced. One interviewee shared that ‘*many disabled people during the [2021] Heat Dome, because of poverty and because of depending on others to survive, were presented with an impossible choice. . .for myself, it would mean me going outside and wheeling to a cooling centre – exerting myself at a time when medical advice is don’t go outside and don’t exert yourself for someone with chronic lung conditions such as mine*’.^49^ Such information from lived experience may not be captured in conventional after-action reports based on healthcare usage. Although the scope of this study did not directly assess how or why these novel impacts were captured in the media and not documented in other investigations, it may be due to the diverse range of sources journalists draw from, including written references and interviews with the public and experts from a range of fields. As journalists often conduct their reporting as events unfold, they benefit from capturing experiences as they evolve. Conversely, interview-based academic studies generally occur many months after the event and may be influenced by memory and recall bias. Additionally, further investigation into the relationship between the impact areas and themes by specific heat-vulnerable groups could provide important insight into how certain groups were affected (e.g. impacts on physical health or disruption of critical social services) which could support the development of future tailored heat interventions and programming.

We also found that many media articles discussed multiple impacts and often presented them as interconnected. For example, 1 article recounted that ‘*June’s [2021] Heat Dome – which killed 362 people in two days – and the catastrophic fires of July and August no longer feel like singular events. They feel like parts of cascading disasters, one triggering the next – the way the baking heat led to the wildfires that begat the mudslides that cut off entire cities and transportation networks*’.^49^ The literature regularly discusses individual outcomes and co-occurring or compounding climate events. However, post-event analyses rarely address the capacity for intersecting events to amplify the effects experienced across multiple sectors simultaneously.^27,69-73^ Thus, as the quote above illustrates, the 2021 Heat Dome impacted human health and co-occurred with, or was compounded by, other climate events, affecting multiple natural and human systems. Other examples reported by the news media include the high demand for air conditioning, which strained the available electricity supply, resulting in localised power outages and increased work demand for service workers, some of whom then faced heat-related illnesses.^74^ These examples demonstrate that media-based analyses can complement conventional post-event assessment methods. Further, this type of analysis also offers the benefit of capturing ‘in the moment’ perspectives which can support after-action reviews, as well as influence reporting and communications during future heat events.

### Limitations and future studies

This project has some important limitations that could be addressed through similar future investigations. First, we sought to capture a broad range of articles across news agencies and websites to ensure our study was comprehensive. However, in doing so, we acknowledge that each source contained its own potential biases^54^ because articles reflect different reporting practices, interests in publishing on extreme heat or climate change, the bias of sources or interviewees and limitations in capacity, such as the ability to have a climate journalist on staff and resources for contacting expert sources.^75^ As such, our findings reflect the interpretations of these sources as content generators. For example, we could not assess how each journalist or news agency decided which topics to prioritise. Because we intended to broadly explore how the impacts of the 2021 Heat Dome were framed and presented to the public, it was not within the scope of this analysis to specifically examine how content differed between media sources or how they approached the inclusion or exclusion of content. Therefore, the findings should be interpreted in the context of these potential influences. Further, we recognise that media coverage may not include equity-deserving groups and may have a bias towards particular groups or markets, among other factors.

Additionally, we did not specifically analyse or attempt to verify the articles for accuracy or validity. Our intention was to investigate the use of media as a strategy that public health and safety officials could use as a supplementary approach following heat events to understand their direct, cascading and compounding impacts. Subsequent studies could help further validate the use of this method by retroactively verifying information published within EHE media articles by impact area with other data sources post-event. Further, this analysis focussed on traditional online media and did not consider social media posts or forums such as blogs. Social media posts and online blogs are often used by public health and emergency preparedness officials and related non-governmental agencies to communicate with the public during EHEs and other extreme weather events. As well, social media is often used by the public during natural hazards and may reflect their experiences and personal impacts.^76,77^ Therefore, future investigations could explore the role of social media in climate communication within Canada. Lastly, it is important to reiterate that the scope of this analysis was limited to media articles specifically published in Canada and addressing the impacts of the EHE within the country. As such, the findings may not reflect the impacts on the various regions affected by the same EHE in the United States. However, this study provides an approach that could be replicated in the United States – perhaps providing the basis for a comparative analysis of impacts between Canada and the United States – or applied to future heat events elsewhere.

## Conclusion

Thousands of news articles on the 2021 Heat Dome were circulated in the media in Canada. We systematically reviewed and qualitatively analysed the content of this large, digitised media dataset and identified the socio-economic impacts of the 2021 Heat Dome. This approach to studying the impacts discussed in the media demonstrates the far-reaching effects of the 2021 Heat Dome on multiple sectors. We found critical consequences for the natural environment, including mass mortality of animals and devastation to vegetation and natural resources. We highlighted extensive effects on social infrastructure and services, from transportation service interruptions to community event cancellations and waste management disruptions. The articles showcased the death toll, as well as immediate and long-lasting physical and mental health outcomes from the heat. Critical infrastructure effects were also observed, including water and electricity supply shortages and major distributions to food producers, processors, distributors and retailers. Lastly, the findings demonstrated diverse repercussions on the private sector, including work cancellations, stoppages, delays, or modifications to over 60 occupations and workplaces. Our results confirmed many of the results of other post-event analysis approaches, as well as identified unique effects not typically captured in after-action reports or the peer-reviewed literature. Thus, a media-based approach to post-event analysis can supplement and complement conventional impact analyses.

## Supplemental Material

sj-docx-1-ehi-10.1177_11786302241276669 – Supplemental material for Media-Based Post-Event Impact Analysis of the 2021 Heat Dome in Canada Supplemental material, sj-docx-1-ehi-10.1177_11786302241276669 for Media-Based Post-Event Impact Analysis of the 2021 Heat Dome in Canada by Emily J Tetzlaff, Nicholas Goulet, Melissa Gorman, Gregory RA Richardson, Paddy M Enright, Sarah B Henderson and Glen P Kenny in Environmental Health Insights
