# Supplementary material for: Media-Based Post-Event Impact Analysis of the 2021 Heat Dome in Canada
Source: Environ Health Insights. 2024 Sep 5;18:11786302241276669. doi: 10.1177/11786302241276669 (PMC11378224; doi:10.1177/11786302241276669)
Supplement: sj-docx-1-ehi-10.1177_11786302241276669 – Supplemental material for Media-Based Post-Event Impact Analysis of the 2021 Heat Dome in Canada [file sj-docx-1-ehi-10.1177_11786302241276669.docx]

**Table S1.** As reported by the media, the direct, cascading, and compounding impacts of the 2021 Heat Dome related to the natural environment (*n* = 1366) in Canada.

| **Themes** | **Concepts** | **Illustrative Textual Examples** |
| --- | --- | --- |
| Natural Systems (*n* = 929) | Wildfires (*n* = 901), Weather Systems (*n* = 89), Vegetation  (*n* = 13) | *“When you add 40-degree heat on top of that the forest will dry out very quickly, making everything in the forest very flammable.” ^(Ref. 1)^* |
| Natural Resources (*n* = 474) | Water (*n* = 280), Air (*n* = 225), Metals & Minerals (*n* = 3) | *“Extreme snow melt is happening on mountain tops due to the high temperatures.” ^(Ref. 4)^* |
| Animals  (*n* = 457) | Pets (*n* = 163), Farm Animals (*n* = 135), Wild Aquatic Animals  (*n* = 130), Insects and Arthropods (*n* = 59), Land Animals (*n* = 55), Birds (*n* = 26) | “T*he toll included six rabbits and three dogs that died at home, as well as one cat and two dogs that had to be euthanized, and two dogs and one rabbit in hospital.” ^(Ref. 7)^* |

*Note: For our analysis, coded data extracts (e.g., words, sentences, paragraphs) were assigned to as many different impact areas and concepts as necessary to describe them. As such, the count values presented may not equal/or may exceed the summative total in which it is nested. n, number of media reports. Ref, reference article within dataset.*

**Table S2.** As reported by the media, the direct, cascading, and compounding impacts of the 2021 Heat Dome related to social infrastructure (*n* = 1121) and services in Canada, as reported by the media.

| **Themes** |  | **Concepts** | **Illustrative Textual Examples** |
| --- | --- | --- | --- |
| Healthcare (*n* = 862) |  | Public Health (*n* = 512), Pre-Hospital Care Services (*n* = 384), Coroner’s Services (*n* = 139), Hospital Service  (*n* = 80), Long-Term Care (*n* = 8), Pharmacies (*n* = 3), Home Healthcare  (*n* = 3) | *“As emergency departments are experiencing an increase in those accessing care for heat-related conditions, [Vancouver Coastal Health] is advising residents to choose the right care at the right place.” ^(Ref. 13)^* |
| Transportation Services (*n* = 143) |  | Railway and Light-Rail Systems (*n* = 42), Public Transit (e.g., City Buses) (*n* = 18), Personal Vehicles (*n* = 7), Ferry Services and Barges (*n* = 1), Flights (*n* = 1) | *“Canadian Pacific says in a statement that mainline operations resumed Monday after safety inspections were completed of the tracks and infrastructure. The company says it is increasing inspections of its tracks and equipment during this period of extreme weather and staff are on-site supporting local authorities. The fire disrupted critical transportation services for the movement of goods across Western Canada and the rest of the continent, CP said.” ^(Ref. 20)^* |
| Education (*n* = 112) |  | Elementary, Secondary and Post-Secondary School/Campus Closures or Modifications (*n* = 96), Cancellation of School Bus Services (*n* = 14), Use of School as Cooling Stations and Strategies (*n* = 13), Mental Health Impacts to Students, Families and Teachers (*n* = 10), Changes to Graduation and Year-End Celebrations (*n* = 9), Use of Online Schooling Platforms (*n* = 1) | *“the schools aren't designed for that heat, and even with air-conditioning on, classrooms could still exceed 30 degrees.” ^(Ref. 21)^* |
| Community and Social Supports  (*n* = 98) |  | Climate Evacuee Support (*n* = 14), Community Centres (*n* = 9), Charity Events (*n* = 8), Senior Support Services, (*n* = 7), Drug User Supports (*n* = 4), Community Meetings (*n* = 2), Environmental Restoration Groups (*n* = 2), Homelessness Support Services (*n* = 49), Memorials (*n* = 2), Mutual Aid (*n* = 2), Churches (*n* = 1), Pet Support (*n* = 1), Sponsorship Programs (*n* = 1), Supporting First Responders (*n* = 1) and Women’s Centers (*n* = 1) | *“Shelters like the Mustard Seed and Alpha House proactively asked the community for bottled water ahead of the scorching temperatures, and the community answered.” ^(Ref. 22)^* |
| Sports & Recreation (*n* = 88) |  | Leisure Activities (e.g., Biking, Golfing, Gambling, Picnicking) (*n* = 27), Fitness and Recreational Activities/Facilities (*n* = 27), Water Activities (*n* = 21), Backcountry Activities (e.g., Camping, Hiking, Climbing, Bouldering, Fishing) (*n* = 20), Park Access (*n* = 17), Sports (e.g., Recreational, Professional) (*n* = 12), Child/Youth Camps (*n* = 4), Indoor Activities (*n* = 3), Cottaging and Resorts (*n* = 2) | “*staff [in long-term care homes] were also canceling outdoor activities to keep seniors safe.” ^(Ref. 23)^* |
| Arts, Culture & Tourism (*n* = 77) |  | Community Concerts and Events (*n* = 26), Tourism (*n* = 23), First Nations Cultural Practices (*n* = 15), Museums and Heritage Sites (*n* = 12) | *“Sorry, Music in the Park fans, it is just too hot to host the hottest ticket in town! With persistent soaring temperatures, the City of West Kelowna will reschedule.” ^(Ref. 24)^* |
| Crime, Corrections & Justice  (*n* = 27) |  | Formalization of Lawsuits (*n* = 8), Assemblies, Rallies, and Protests (*n* = 7), Climate Justice (*n* = 6), Courthouse Closures (*n* = 2), Looting Warnings (*n* = 2), Eviction Notices (*n* = 1), Arrests (*n* = 1) | *“the Pacheedaht First Nation is asking protesters to vacate the Fairy Creek area and other forested areas during the extreme heat. The nation said it's worried about the possibility that human-caused wildfires could threaten the Pacheedaht community.” ^(Ref. 25)^* |
| Waste Management (*n* = 12) |  | Curbside Collection (*n* = 9), Wastewater and Sewage Infrastructure (*n* = 2), Depot Closures (e.g., Landfills, Recycling Depots) (*n* = 1), Wildfire Hazard Vegetation Debris Disposal (*n* = 1) | *“Due to the extreme high temperatures, and concerns for the health and safety of collection crews, please expect delays or cancellations with curbside collection this week.” ^(Ref. 26)^* |

*Note: For our analysis, coded data extracts (e.g., words, sentences, paragraphs) were assigned to as many different impact areas and concepts as necessary to describe them. As such, the count values presented may not equal/or may exceed the summative total in which it is nested. n, number of media reports. Ref, reference article within dataset.*

**Table S3.** As reported by the media, the direct, cascading, and compounding impacts of the 2021 Heat Dome related to human health (*n* = 1074) in Canada.

| **Themes** | **Concepts** | **Illustrative Textual Examples** |
| --- | --- | --- |
| Mortality  (*n* = 861) | Heat-Related Fatalities  (*n* = 861) | *“As a result, 595 people perished from B.C.’s unprecedented and record-setting heat, according to a government report. The fatalities were reported to the BC Coroners Service between June 18 and Aug. 12, 2021. In that period of time, the largest number of deaths occurred from June 25 and July 1 during a heat dome event.” ^(Ref. 27)^* |
| Physical Health  (*n* = 219) | Heat-Related Injury (*n* = 160), Opioid-Related (*n* = 23), Water Safety  (*n* = 22), Fall from Heights (*n* = 6), Food Spoilage  (*n* = 6), Burns (*n* = 4), Mudslides & Floods (*n* = 2) | *“Over the weekend, with temperatures hitting the mid-40s in some places, paramedics responded to more than 200 cases recorded as heat-related illness in just two days -14 times the number logged over the entire month of June last year.” ^(Ref. 28)^* |
| Mental Health  (*n* = 183) | Other Mental Health Impacts (*n* = 67), Anxiety & Depression  (*n* = 57), Frustration & Stress (*n* = 40), Mental Health Support Services (*n* = 28), Eco-Anxiety & Climate Grief  (*n* = 18), Trapped & Isolated (*n* = 3) | *“Extreme heat waves are leading to an unprecedented death toll across the province. Not as severe, but nonetheless significant, these conditions are negatively affecting our physical and mental health, disrupting our ability to work, study, and sleep.” ^(Ref. 35)^* |

*Note: For our analysis, coded data extracts (e.g., words, sentences, paragraphs) were assigned to as many different impact areas and concepts as necessary to describe them. As such, the count values presented may not equal/or may exceed the summative total in which it is nested. n, number of media reports. Ref, reference article within dataset.*

**Table S4.** As reported by the media, the direct, cascading, and compounding impacts of the 2021 Heat Dome related to critical infrastructure (*n* = 988) in Canada.

| **Theme** | **Concepts** | **Illustrative Textual Examples** |
| --- | --- | --- |
| Food Systems (*n* = 356) | Field Crop Farming (*n* = 289), Gardening (*n* = 54), Supply Chain and Food Security (*n* = 34) | *“The floods are disrupting supply chains. Shelves in food stores in the interior of the province are already being emptied. Goods that would normally be leaving the Port of Vancouver headed to Alberta and points east are going nowhere. A large percentage of the province’s dairy and poultry production has been lost. There will be more empty shelves.” ^(Ref. 42)^* |
| Community Water Supply (*n* = 159) | Water Supply and Access (*n* = 135), Water Advisories (*n* = 26), Water Consumption and Demand (*n* = 26) | *“The record high temperatures in Old Crow came after three weeks of extraordinarily low water levels in the Porcupine River. Benoit Turcotte, senior researcher in hydrology and climate change at Yukon University,*  *explained that this year marks the lowest level ever recorded in a 46-year tracking period for the Old Crow River, which flows into the Porcupine River from Old Crow flats.” ^(Ref. 43)^* |
| Energy  (*n* = 152) | Energy Consumption and Demand (*n* = 121), Power Outages (*n* = 26), Infrastructure Damage (*n* = 19), Disrupted Maintenance and Suspensions (*n* = 9), Economic Challenges (*n* = 9) | *“The heat wave also stressed energy infrastructure as power grids worked to keep up with demand.” ^(Ref. 44)^* |
| Transportation Infrastructure (*n* = 105) | Roadways (*n* = 94), Sidewalks (*n* = 13) | *“These weather changes were felt by the entire province of B.C. as 2021 saw massive heat wave events and torrential downpours of rain that flooded the province’s infrastructure and caused mass destruction to the highway system.” ^(Ref. 45)^* |
| Communications (*n* = 7) | Tower Damage (*n* = 3), Disrupted Service (*n* = 3), Mail Services (*n* = 2) | *“Telus Corp. says its has deployed emergency communications equipment to support local authorities and emergency crews dealing with the wildfire in Lytton, B.C.” ^(Ref. 46)^* |
| Commercial Buildings (*n* = 1) | Exploding Glass (*n* = 1) | *“You saw those photos perhaps from Squamish, where there were tempered glass panels on people’s desks that just kind of exploded in the heat yesterday.” ^(Ref. 47)^* |

*Note: For our analysis, coded data extracts (e.g., words, sentences, paragraphs) were assigned to as many different impact areas and concepts as necessary to describe them. As such, the count values presented may not equal/or may exceed the summative total in which it is nested. n, number of media reports. Ref, reference article within dataset.*

**Table S5.** As reported by the media, the direct, cascading, and compounding impacts of the 2021 Heat Dome related to the private sector (*n* = 165) in Canada, as reported by the media.

| **Theme** | **Concepts** | **Illustrative Textual Examples** |
| --- | --- | --- |
| Business (*n* = 165) | Restaurants (*n* = 45), Air Conditioning Distributors (*n* = 43), General Retail Stores (*n* = 32), Hotels & Hospitality (*n* = 31), Mining, Oil & Natural Gas (*n* = 18), Farmers Market Vendors (*n* = 11), Christmas Tree Distributors (*n* = 10), Insurance Companies (*n* = 6), Nurseries & Garden Centres (*n* = 6), Grocery Stores (*n* = 5), Housing Market (*n* = 4), Forestry (*n* = 4), Gasoline (*n* = 2), Recreational Companies (*n* = 2), Banks (*n* = 1), Cannabis Distributors (*n* = 1), Roofing (*n* = 1), Tanning Salon (*n* = 1) | *“But this unusual 'heat dome,' which is a strong ridge of high pressure that traps warm air, is also pushing some companies to cut operating hours and actually take the more dramatic step of suspending business until it subsides.” ^(Ref. 48)^* |

*Note: For our analysis, coded data extracts (e.g., words, sentences, paragraphs) were assigned to as many different impact areas and concepts as necessary to describe them. As such, the count values presented may not equal/or may exceed the summative total in which it is nested. n, number of media reports. Ref, reference article within dataset.*
